# Visual attention outperforms visual-perceptual parameters required by law as an indicator of on-road driving performance

**DOI:** 10.1371/journal.pone.0236147

**Published:** 2020-08-14

**Authors:** Wolfgang Grundler, Hans Strasburger

**Affiliations:** 1 Volkswagen AG, Wolfsburg, Germany; 2 Department of Medical Psychology and Medical Sociology, Georg-August-Universität Göttingen, Göttingen, Germany; 3 Institute of Medical Psychology, Ludwig-Maximilians-Universität München (LMU Munich), Munich, Germany; Universitat de Valencia, SPAIN

## Abstract

**Purpose:**

A variety of visual and psychometric tests have been developed for assessing on-road driving performance and fitness to drive. The diagnostic power of a state of the art psychometric test battery (Vienna Test System) combined with a set of standard visual parameters recommended for assessing fitness to drive is investigated using an on-road driving test. The study aimed to determine whether a psychometric test battery could predict older adults’ on-road driving performance. The relevance of visual standards required by law is discussed.

**Methods:**

Vision impairment is more prevalent in later adulthood and many studies on visual and cognitive impact on driving safety and performance therefore focus on adults above 60 years of age. We therefore acquired an extensive set of driving-related visual and psychometric performance parameters in a group of elderly drivers (N = 84, median age 69, SD 6.6 years). Visual assessment included foveal acuity, perimetric field size, and dynamic aspects of peripheral vision (termed “PP”) in the computer-based Vienna Test System (VTS; Schuhfried), as well as letter contrast thresholds in foveal and parafoveal vision in a separate setup. A selection of psychometric driving-aptitude tests that demonstrated the battery’s capacity to predict aspects of driving performance and safety were further conducted on the VTS. Driving performance was assessed in a standardized on-road driving test. Two independent observers rated driving performance using a fixed scoring system assessing the number of driving errors in pre-defined traffic situations. In addition, globalized driving competence scores were assigned on a 6-point scale.

**Results:**

The test battery performed excellent in identification of good drivers but failed in the prediction of bad driving performance. Visual performance indicators required by German law were less indicative of driving ability than psychometric assessment. Selective and divided attention turned out to be much more important for predicting fitness to drive than either visual acuity, size of the visual field, or contrast sensitivity.

**Conclusion:**

Predicting fitness to drive by means of visual and psychometric tests is an ambitious challenge. On the one hand sensitivity of a multi-disciplinary test-battery is too low to predict reliable driving ability in diagnostic settings which require an unambiguous interpretation of test results for individual drivers. Low sensitivity and low predictive values are incompatible with that objective. On the other hand, the results are valuable for a routine screening of fitness to drive. For that case, the assessment of attentional abilities in particular appears to be promising. Performance measures of divided and selective attention showed themselves to be the most predictive for fitness to drive in a sample pre-screened for clear visual deficits. Visual performance parameters required by law, in contrast, had no meaningful impact on driving performance, indicating a gap between mandatory regulations of state authorities and research results. Our results suggest that visual acuity tests designed for clinical diagnosis and monitoring of eye diseases should not at all be the choice for a screening of fitness to drive.

## Introduction

Driving is a multifactorial competence. Driving means to enact distinctive, driving-specific competences, like navigation and orientation, lane keeping, speed control, distance monitoring, environmental scanning, giving way [1, 2]. A certain level of basic sensory and psychological functions like visual and attentional capabilities further have a profound impact on driving-specific performance requirements. [1]

Unlike driving competence, *fitness to drive* is a legal term that is defined by respective national legislation. In the European Union’s member states, for example, there were about 110 different driver license models in 2006, with differing rights of disposal and periods of validity [3]. Requirements for fitness to drive now differ mainly depending on vehicle class. According to Annexe III of the EU Directive on driving licences, two groups of vehicles are distinguished: Group 1 comprises motorbikes, passenger cars, and tractors for agriculture and forestry (license classes A, A1, A2, B, BE, AM, L, and T), and Group 2 comprises trucks, truck trailers above 750 kg total mass, busses, and passenger transportation (license classes C, C1, CE, V1E, D D1, DE, D1E).

Based on that EU Directive, the German Driving Licences Regulation [4] entitles permanent residents aged 18 and above posessing verified visual abilities and knowledge of regulations to obtain a car driving license in Germany (Group 1). Adequate driving competence must be confirmed by an independent observer (examiner) in a driver’s license examination. Psychological areas of competence (stress load capacity, navigation skills, ability to concentrate, attentiveness, responsiveness) are not routinely checked and are only tested as warranted (at indications of degraded performance or traffic offences).

Basically, assessment of driving fitness rests on a categorical diagnostic model: fitness (the criterion) is inferred from the scores on a pre-defined set of performance measures, the (preconditions). In the subsequent dichotomization, individuals are classified as fit, or unfit, to drive [5]. Diagnostics for fitness to drive follow a two-part strategy of psychological testing and visual assessment.

There are degrees of freedom in designing the diagnostic model’s specifics since no predefined methods are named for assessment of psychological fitness to drive. Psychometric testing of fitness to drive tries to reduce the conceptual framework of driving competence to measurable targets [6]. Assessment is therefore, in practice, based on individual performance indicators that have shown predictive validity for fitness to drive in standardized driving tests [7–10]. In Germany, computerized test batteries consisting of specific test procedures for assessing visual orientation (i.e. perceptual speed in analyzing pictures of traffic scenes), selective attention and concentration (i.e. ability to select and respond to a predefined stimulus in a set of distractors), reaction-time (i.e. measuring simple choice reaction time to a binary set of auditory and visual stimuli), and stress-related cognitive-physical responsiveness (i.e. performance measures in complex multiple-stimuli and multiple-response settings affording quick and correct reactions) have been evaluated, not least because of these abilities being legally required by German driving license regulations [11].

Current procedures for psychometric testing of fitness to drive–the Expert System Traffic (XPSV) (on the Vienna Test System; Dr. Schuhfried GmbH), the ART 2020 (Austrian Road Safety Board (KfV), Vienna), and the TAP-M (Psytest Psychological Test Systems)–have been evaluated for being engineered in accordance to scientific standards and providing validity concerning aspects of traffic safety in a research project conducted by the German Federal Highway Research Institute (FE 82.291/2005) [12]. All of these systems were shown to provide valid results for predicting fitness to drive. Hence the Vienna Test System is one of three commonly used state of the art systems for testing fitness to drive in Germany. The Vienna test system is the most often evaluated system in the field of traffic psychology. Google Scholar shows more than 1.500 matches for the term “Vienna Test System”, 412 matches for “ART 2020”, and 143 matches for “TAP-M” (transcribed as “Testbatterie zur Aufmerksamkeitsprüfung Mobilität”). A strong point of the VTS is the standardization of tests, accompanied with documentation and the availability of test quality measures, in particular reliability and validity. For certifying adequate psychological fitness, relative scores, measured against an age-independent standard sample, are defined. According to recommendations of the German Federal Highway Research Institute (BASt), the requirements are deemed as met if a percentile rank of 16 is attained (or surpassed) in all of the employed psychometric performance tests [12].

In the U.S., a similar research project, conducted by the National Highway Traffic Safety Administration (NHTSA, DOT HS 809 581), studied the feasibility as well as the scientific validity and utility of performing functional capacity screening with older drivers [13]. The relationship between perceptual-cognitive and physical abilities and a number of traffic-safety outcome measures (crash-involvement and moving violations, such as running a stop sign or traffic signal, failure to yield, one-way and wrong-way violations, etc.) had been analyzed. A set of guidelines for the American Association for Motor Vehicle Administrators (AAMVA) was suggested in the report. The screening test battery addressed perceptual-cognitive abilities and included the Motor-Free Visual Perception Test/Visual Closure subtest (a test for assessing visuo-spatial skills, including the ability to visualize missing information as needed when only part of a critical target is visible to a driver); the Trail-Making Test, Part B (a paper-and-pencil exercise to measure directed visual search and divided attention capabilities); Dynamic Trails (a PC-based assessment of directed visual search and divided attention abilities with an added element of distraction provided by a moving traffic scene in the background); the Useful Field of View (UFOV), Subtest 2 (PC-based measurement of divided attention and information processing speed), and some procedures addressing physical abilities, i.e. the Rapid Pace Walk and Foot Tap tests (which measure lower limb strength and mobility as required to sustain steady control over brake and accelerator, and to quickly shift from one pedal to the other as circumstances may require); Head/Neck Rotation, and Arm Reach [13]. Among the perceptual-cognitive abilities, the Motor-Free Visual Perception Test/Visual Closure subtest was most predictive for identifying at-risk drivers by a wide margin. Three additional perceptual-cognitive measures—Trail-making (Part B); Delayed Recall; and Useful Field of View (subtest 2)—were also shown to be potentially useful predictors. Among the physical measures, the Rapid Pace Walk and Head/Neck Rotation appear to have the greatest potential value as predictors of driving impairment. Interestingly, absolute (rather than relative comparisons) cut-off values were defined for either indicating the need for prevention efforts or intervention have been calculated, i.e., for the UFOV (Subtest 2) a value of 200 msec (prevention) and 300 msec (intervention).

In Australia, Wood et al. developed a very similar, multi-disciplinary driving assessment battery, incorporating tests from vision, cognitive, and motor domains [14]. Central motion sensitivity was measured using a computer-based random dot kinematogram (Dot Motion) [15], further test were a computerized choice-reaction-time task (Colour Choice Reaction Time) [14], Postural Sway (participants standing on a medium-density foam mat with their eyes closed are asked to remain as still as possible for a 30 second duration) and kilometers driven on average per week were recorded.

In contrast to the relative-measures principle of psychometric psychological testing described above (where results are expressed as relative scores, set in relation to age-independent standard samples) tests of visual assessment from the ophthalmic and optometric tradition provide sample-independent, physically defined cut-off values for testing fitness to drive (Table 1). Essential for defining visual performance requirements in Europe are the basic recommendations of the EU’s Eyesight Working Group [16]. According to the guidelines, binocular visual acuity should be 0.3 logMAR or better. The field should have a horizontal extent of at least 120 degrees. It should furthermore extend to 20 degrees above and below the horizontal meridian, and a minimum of 50 degrees to the right and to the left are suggested. There are no requirements for twilight vision (like low-light contrast sensitivity or glare sensitivity), other than in cases of doubt. Even though tests for twilight vision could provide useful information about driving capacity (contrast sensitivity in particular has been found to have a stronger relation with traffic accidents and violations than visual acuity), it is not clear what cut-off value and method of measurement to use for contrast sensitivity, nor which cut-off value for glare sensitivity. As the authors emphasize, the rationale for recommended cut-off values have not yet been properly justified.

**Table 1:** Cut-off values for visual assessment.

Visual function	License classes C, C1, CE, C1E, D, D1, DE, D1E (i.a. truck, bus)	License classes A, A1, A2, B, BE, AM, L und T (i.a. motorbike, passenger car)
**Visual acuity (logMAR)**	0.1 / 0.3	0.3 (better eye or binocular)
**Visual acuity in case of monocular vision**	not permitted	0.3
**Permissible spectacle lens**	+ 8.0 D (astigmatic corrections to be substituted by spherical equivalent)	no limitation
**Visual field size**	normal visual fields of both eyes, at least normal binocular visual field	normal visual field of at least one eye or balanced binocular visual field
**Examination of fixation and ocular alignment**	no diplopia permitted within main field of view (25 deg glance up, 30 deg sideways glance, 40 deg glance down), graduated evaluation of binocular vision dependent on vehicle class	muscular strabismus and concomitant strabismus without diplopia within a visual area of at least 20 deg of diameter accepted, normal head posture recommended
**Contrast sensitivity, glare sensitivity**	contrast ratio 1:2.7; required minimum ratio 1:5, otherwise ban on night driving	contrast ratio 1:5; required minimum ratio 1:23, otherwise ban on night driving
**Colour sensitivity**	not permitted: protanomaly (anomaly ratio below 0.5) and protanopia	no requirements

Cut-off values for visual assessment provided by the German Ophthalmologic Society (DOG) and Association of German Opththalmologists [17].

Across countries, in an international comparison strikingly similar standards for certifying fitness for driving are in place for both testing visual sensory function (acuity, visual field, and contrast sensitivity) and methods for their measurement [18]. Part of that consensus might be due to a common point of view when discussing visual performance in connection to causes and consequences of vision loss. In particular, aspects of vision are there typically focused on structural and functional changes of the eye. Cognitive visual skills (like reading, orientation), in contrast, are not discussed [19, 20]. Furthermore, the importance of (non-visual) cognitive skills for safe driving appears to be generally underestimated, in light of the fact that these are not standardly required for a common driver’s license. While there is a consensus about the necessity of visual-cognitive attentional fitness, evidenced e.g. in the studies on the UFOV test [21], studies on the diagnostic quality of cognitive tests for predicting fitness for driving, and in particular their comparison to visual skills for that aim, are rare. Wood et al. showed that a multi-disciplinary driving assessment battery was superior to measures of visual function tests (visual acuity, contrast sensitivity, and visual field) in its ability to predict on-road driving performance outcomes [22].

The purpose of our study was to investigate what aspects of vision and psychological testing are most predictive for an assessment of fitness to drive. Visual acuity was included because it has face validity and represents the exclusively necessary requirement for driving license applicants [23]. Assessment of further visual parameters followed recommendations of the European’s Eyesight Working Group [16]. Procedures of psychological testing as an incident-driven assessment due to reasoned doubts of driver's license authorities about a driver’s fitness to drive have been conducted according to requirements of the German Federal Highway Research Institute [5]. We therefore assumed that visual and psychological abilities should both add equivalent diagnostic value for predicting fitness to drive assessed in an in-traffic on road driving test.

## Materials and methods

### Participants

Participants were 84 older drivers aged 60 years and above (56 = 67% male, 28 = 33% female; aged 60–91 yr with a mean age of 68.9, SD 6.6 yr), recruited by advertisements in local newspapers. Subjects were payed for joining the study. None of the participants reported ongoing ophthalmic treatment or eye disease (detected or declared). However, data were excluded from analysis if the individual dataset showed significant visual field loss that indicated ocular or visual pathway disease, or was simply due to systematic measurement error. In sum, seven participants were excluded from the original sample. Three of them showed significant visual field loss that indicated ocular or visual pathway disease, four participants were excluded due to indications for systematic measurement error. None of the participants was excluded due to insufficient visual acuity required by law (decimal acuity 0.3, for the better eye or binocularly).

All participants were current drivers and licensed to drive in Germany. They were given a full explanation of the nature of the study and experimental procedures, and written informed consent was obtained. The study adhered to the tenets of the Declaration of Helsinki, and has been approved by the Human Science Center (HWZ) as a permanent institution of the University of Munich. Participants attended two testing sessions. The first included a series of tests of vision and psychometric assessment and took about 2 ½ hours. The second session comprised a one-hour on-road driving test.

### Visual assessment

Visual assessment followed the recommendations of the Eyesight Working Group who suggest to use those functions that are based on reasoning, common sense and practical experience important for safe driving [16]. We therefore included measurements of visual field size, visual acuity, and contrast sensitivity (Table 2).

**Table 2:** Visual functions, test devices and test procedures.

Visual function	Test device	Test procedure
**Visual acuity**	Binoptometer (OCULUS, G/59850/0207/d)	Recognition of Landolt-C optotypes varying in size and angular position
**Field of view**	Perimeter (Octpopus 101, Haag-Streit)	Detection of kinetic stimuli moving from the outer boundaries towards the visual field center
**Contrast sensitivity**	R_Contrast (Strasburger, 1987)	Recognition of numerals varying in contrast (light on gray background) presented at one of five predefined locations in the center (0°) and near-periphery (10°)

For diagnosing visual field size, an automated kinetic perimetry was performed (Octopus 101, Haag-Streit, Interzeag, Switzerland; Software PeriTrend V6.05). The OCTOPUS 101 is a 90°-field cupola perimeter. The perimeter was installed in a darkroom and was controlled by a standard personal computer operating under Microsoft Windows. We used the Octopus Goldmann Kinetic Perimetry (GKP) module with a Goldmann III 4e stimulus (stimulus “size III”has a diameter of 0.43°; intensity “4e”corresponds to 1000 asb, i.e. 318 cd/m^2^). The kinetic module allows moving stimuli slowly from the outer boundaries of the visual field towards the center. Along 24 radii separated by an angle of 15 deg, stimuli moving at an angular velocity of 4°/s were presented in random order. Stimuli were preannounced by an acoustic cue each, to ensure the stimulus is attended to. Participants responded by pressing a button, thereby indicating that the stimulus had been detected. The area seen by the respective, steady fixating eye defines the monocular visual field. Binocular visual field size and maximum field size on the horizontal meridian were computed manually. No trial lenses for correction of refractive error were applied. Quality of eye fixation was monitored by the system interrupting the examination when the patient was not fixating or closing the eye. Participants showing characteristic defects that indicated structural damage of the visual system (e.g. acute loss of visual field or quadrantanopia) on the basis of a visual analysis of printouts (analyzing the shape the visual field) were excluded from the study. Isopters were not corrected for participant's reaction time.

Visual acuity was measured using an Oculus Binoptometer (Binoptometer 3, G/59850/0207/d). The device allows visual acuity measurement using standardized optotypes (Landolt C) and standardized viewing conditions (DIN EN ISO 8596). Optotypes are presented in eight orientations. The natural status of the accommodation-convergence cross coupling is maintained by optical means in the apparatus. The simulated free-space viewing conditions thus induce no instrument myopia. To ensure reliable test results the system provides predefined assessment procedures by micro-processor controlled test sequences. For controlling and result-storage an external serial interface was used (RS 232C). Test sequences 4 and 5 of procedure G25 were selected for reporting visual acuity in six predefined steps: 0.30, 0.22, 0.15, 0.10, 0.00, and –0.10 logMAR.

Contrast sensitivity was measured using the routine *R_Contrast*, a program developed for rapid assessment of recognition contrast thresholds [24]. The recognition contrast threshold is defined as the level of contrast needed for correctly identifying a pattern out of a number of alternative patterns. Standard stimuli were the 10 numerals (0 to 9; size: 1° visual angle; presentation time: 100 msec; background luminance: 62 cd/m^2^) presented as light on gray patterns on a 19-inch flat screen. Stimuli were presented singularly in random order at one of five predefined locations in the central visual field. Participants were positioned at a constant viewing distance of 43 cm from the monitor, with distance stabilized by a chin- and head rest. Recognized numerals were reported by keyboard entry. *R_Contrast* uses the adaptive maximum-likelihood technique *ML-PEST* developed by Harvey [25, 26] for stimulus presentation and threshold estimation. Both center-fovea (0°) and near-peripheral (eccentricity 10°) recognition contrast thresholds were obtained.

### Psychological assessment

The Vienna Test System (VTS) that we used for the psychometric assessment is a computerized assortment of tests that can be used singly, or combined as test batteries. Part of the system is the “Expert System Traffic” (XPSV) that has been developed for subject assessment in the field of traffic psychology. At the core of the Expert System Traffic are two standardized test batteries (Standard and Standard Plus) that can be used to test abilities relevant to traffic. Various tests were selected from the XPSV, focusing on perceptual performance in traffic situations, on selective and divided attention, and reactive performance (Table 3) [27, 28]. Psychological testing of older adults took about two hours with large variance (Mean: 110 minutes, SD: 25, range: 73–224). Noteworthy especially maximum test durations exceeded the approximate range of processing times provided by the manufacturer of the system (84–169 minutes).

**Table 3:** Psychological functions and description of test procedures.

Psychological function	Test designation	Test procedure
**Intelligence**	Adaptive Matrices Test (AMT; Form: S01; Version 23.50)	**Task**: selection of one of eight response items that supplements a matrix of nine elements, based on explicit construction rules for logical reasoning
		**Resulting measure**: general intelligence
**Learning ability**	Non-Verbal Learning Test (NVLT; Form: S02 short A; Version 21.11)	**Task**: nonverbal stimuli (100 geometrical patterns) are successively presented from which 8 are repeated a total of 5 times; stimuli that already appeared are to be remembered and reported
		**Resulting measure**: number of correct and incorrect answers, number of differences between correct and incorrect answers
**Visuo-motor coordination**	Double Labyrinth Test (B19; Form: exclusive; Version 24.00)	**Task**: maintaining two markings (circles) in the middle of two tracks (lanes) by means of two control knobs, without touching the tracks’ edges
		**Resulting measure**: frequency and duration of touching the edges
**Perceptual performance in traffic situations**	Tachistoscopic Traffic Test (TAVTMB; Form: S01; Version 27.03)	**Task**: identifying which of a number of traffic-relevant elements were part of static traffic situations (photographs) shown for 1 sec on a screen
		**Resulting measure**: number of correctly and incorrectly identified elements
**Reactive performance**	Vienna Determination Test (DT; Form: S01; Version 31.01)	**Task**: reacting to multiple visual-auditive stimuli in an adaptive multitask test scenario, becoming increasingly stressful the better the subject performs
		**Resulting measure**: number of correct reactions to visual-auditive stimuli reported by keypad and footpad
**Reaction time**	Vienna Reaction Test (RT; Form: S03; Version 27.11)	**Task**: reacting to given critical constellations of visual and auditory stimuli by moving a finger from a resting position (resting button) to a response button
		**Resulting measure**: reaction time (split into reaction time and motor time)
**Selective attention**	Cognitrone (COG; Form: S01; Version 34.00)	**Task**: comparison of patterns with regard to their congruence
		**Resulting measure**: sum and mean time (sec) of correct rejections of non-congruent stimuli
**Selective attention**	Signal Detection (SIGNAL; Form: S02; Version 25.01)	**Task**: visual discrimination of critical stimuli in a continuously changing dot cloud presented on a screen
		**Resulting measure**: median time (sec) needed for registration of critical stimuli (four dots forming a square)
**Divided attention**	Peripheral Perception Test (PP DEV; Form: exclusive; Version 21.11)	**Task**: tracking of a moving stimulus presented in the central visual field by turning a rotary knob
		**Resulting measure**: frequency and duration of mistakes on a central tracking task in a dual task scenario (requires divided attention between a central tracking tasks and a peripheral detection task)
**Peripheral detection**	Peripheral Perception Test (PP FOV; Form: exclusive; Version 21.11)	**Task**: detection of visual stimuli presented on the horizontal meridian in a dual-task scenario (requires attention to be divided between a central tracking task and a peripheral detection task)
		**Resulting measure**: size of the useful field of view on the horizontal meridian

Psychological functions and description of test procedures of the Vienna Test System (VTS), used for psychometric assessment.

### Driving assessment

Driving performance was assessed in a standardized on-road driving test in natural traffic environment representing a typical setting for driver licensing tests in Germany. Open-road test designs are known to have particularly high validity and represent the gold-standard for assessing driving performance [29]. Driving assessment for the study took place in a car from a driving school (Audi A3) with manual gearshift. An accredited, professional driving instructor sitting in the front passenger seat was responsible for directing the driver along the route and monitoring safety. Whenever the situation allowed, informal conversation was being made while driving to relax drivers and to distract from the stated topic of having a driving a test–unless drivers did not wish to do so. Upon finishing the route, subjects were welcome for self-assessment and feedback from the driving instructor.

Participants drove along a 20-km route on the open road, starting with a short familiarization period. The driving assessment took around 60 minutes to complete. It was conducted either at mid-morning or mid-afternoon. The route included different kinds of road in the city, along suburban streets and two-lane bypass roads, and on small highways without centered road marking. The range of driving behaviors included *merging* (lane changing, merging and entering/exiting traffic flow), *priority/giving way* (at intersections, pedestrian crossings, and roundabouts), behavior at traffic-light controlled intersections, as well as traffic sign recognition for orientation and navigation. It thus complied with typical settings for driving assessment [30, 31].

Driving was scored both by the driving instructor and by a psychologist trained in test drives, seated in the back of the car (backseat evaluator). Scoring was done at 52 predefined sections along the route. At each of the 52 sections, relevant observational aspects of driving were evaluated in four categories (0 = not observable; 1 = mistake; 2 = solved; 3 = very well solved). In sum there were 134 observations per driver. Each observation was further assigned to one of nine behavioral categories: *distance behavior* (keeping appropriate distance to vehicles in front and to the side, and to other road users), *lane keeping* (lane departures, lane positioning in curves, driving in the middle of the lane), *transparent communication and interaction* (non-verbal communication to other road users and pedestrians to indicate one’s own behavioral intentions; i.e. face-to-face interaction and manual signaling), *speed behavior* (exceeding speed limits, inappropriate speed; i.e. driving too slow in relation to other drivers; speed compromising safety), observation of blind spots (correct checking for blind spots and shoulder checks, checking the rear-view and side mirrors, having a second view at intersections), *priority/giving way* (give way to the right, give way at intersections), *indicating/signaling* (appropriate use of the directional indicator) *attentive and perceptive behavior* (scanning the environment, paying visual attention to other road users, pedestrians, and bicycles) and *anticipatory driving* (avoiding heavy accelerations and decelerations or short intervals between accelerating and braking, appropriate planning and preparation). The proportion of errors observed by either the driving instructor or the psychologist was calculated for each observational category.

In addition, overall driving performance was scored on a 6-point scale based on driving standards criteria. Driving scores from 1 to 6 were assigned as subjective ratings that reflect the error scores obtained during the test drive. Consistency of these results was checked by rank correlations (Spearman Rho) between the two scoring systems (number of mistakes observed, and globalized ratings). Assessments showed themselves to be highly consistent, in particular for those categories that were easy to observe (like lane keeping, speed behavior, observation of blind spots). Higher proportion of errors led to lower globalized ratings. In line with our consistency checks, globalized driving scores were previously shown to be valid measures for differentiation between good and bad driving performance [22, 32–34]. A score of “1” indicated excellent driving skills with near flawless behavior. Drivers scoring “2” would definitely pass the licensing test, indicating average driving skills with minor driving errors. A score of “3” indicated below-average driving and observation skills, where the driver might or might not pass the licensing test. There are, however, no major driving errors. A score of “4” indicated the driver would definitely fail the licensing test. Drivers with that score failed to drive in a safe manner; major driving errors had been observed, traffic rules had been disregarded. Scores of “5” or “6” indicated that drivers represent an increased risk for other road users or that the instructor had to take action to avoid an incident. Overall driving performance was then calculated as the mean value of the ratings from both observers, instructor and psychologist. The driving instructor was not informed about the participants’ functional performance in the laboratory testing; the back-seat evaluator conducted both the laboratory testing and driving assessment, for practical reasons. Inter-rater reliability for the ratings between the driving instructor and backseat evaluator was excellent, with an inter-rater correlation coefficient (Spearman Rho) of 0.86 (p<0.001).

### Statistical analysis

For the statistical analysis, in a first step drivers were grouped, post-hoc, as *safe* or *unsafe*, according to their overall driving performance scores. Drivers being scored below 2.5 (indicating driving skills where the driver may fail the licensing test) were labeled “possibly unfit to drive”, and drivers who scored 2.50 or better were labeled “fit to drive”. Hence, the chosen cut-off value of 2.5 represents a very strict and rigid criterion. It was chosen to suit the categorical diagnostic model that is commonly used for testing driving ability, resulting in a split between drivers fit to drive or the opposite. Bivariate diagnostic models, by definition, cannot differentiate performance measures in between safe and unsafe. We thus had to decide between a strict and a tolerant bivariate model. Due to the statistical requirements for balanced samples sizes, we decided in favor of the strict model. Since “unfit to drive” might be a misleading label for drivers that scored around 3 in the driving test we described them as “possibly unfit to drive”.

Post-hoc group differences for vision and driving characteristics were examined using independent t-tests and χ^2^ tests, where appropriate. When group differences were significant, effect sizes were calculated using an online analysis tool provided by www.psychometrica.de [35], as significance and p values on their own are insufficient and can be misleading in interpreting data [36–38]. As interpretation of effect sizes depends on context, we interpreted the practical relevance of effect sizes using recommendations of J. Ferguson for social science data [36]. In particular, Ferguson proposes a minimum size representing a “practically” significant effect for social science data (“RMPE”). We will interpret effect sizes above that value but below the moderate size as ‘small effect’.

In a second step, for each subset of variables (visual and psychometric measures), multiple regression with backward elimination via the Wald criterion (alpha = 0.1) was used, to isolate those variables with most power for predicting driving performance. Using the derived subset of visual and psychometric predictors, we attempted to reproduce the groups by means of binary logistic regressions. Data were analyzed with SPSS (V. 19.0; www.ibm.com).

## Results

**Fig 1:**
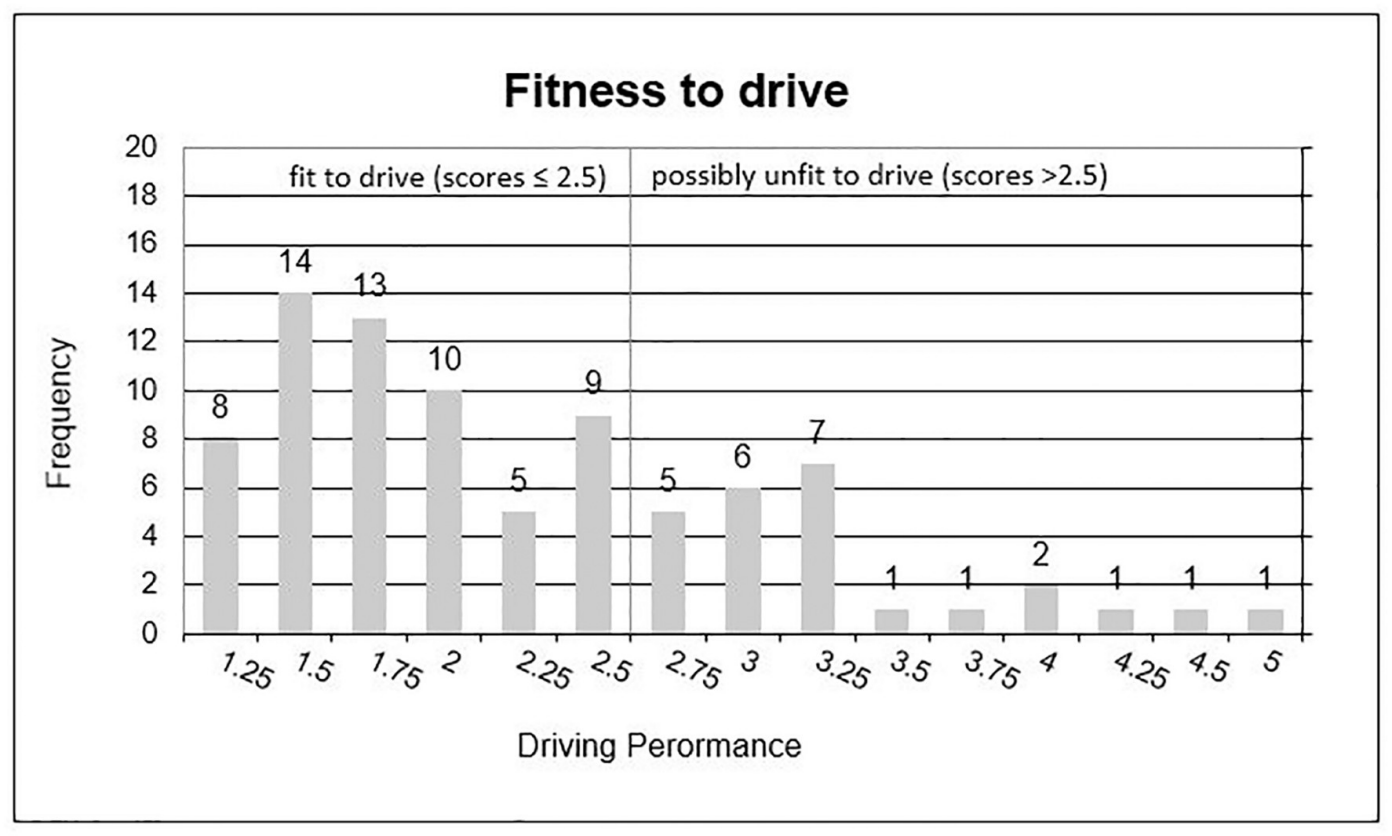
Participant’s driving performance participants. Drivers were grouped post-hoc based on their overall driving perfcormance scores. A cut-off of 2.5 was chosen for indicating those drivers who might fail a licensing test (labeled “Possibly unfit to drive”).

The sample was grouped into “fit to drive” (n = 59) and “possibly unfit to drive” drivers (n = 25) based on their driving performance in the on-road driving test described in Statistical analysis (Fig 1). In total, 59 drivers (70%) were assessed to definitely meet the driving license requirements for standard on-road driving tests (scores 1 and 2), while, on the other side, 7 drivers (8%) were assessed to definitely fail driving in a safe manner (scores 4 and 5; risky drivers). Driving scores in between (around 3) were labeled as below-average drivers (18 drivers, i.e., 21%). So, very bad drivers were rare; the majority of older adults in the study were, on the whole, good drivers.

Table 4 shows driving characteristics of results indicating safe and potentially unsafe drivers. Not surprisingly there are striking differences between the post-hoc groupings of the sample, evidenced by mostly substantial effect sizes in nearly all observed categories. In particular deficits in give way situations, missing observation of blind spots, inappropriate speed behavior, and missing attentive/perceptive behavior mark drivers with low driving competence (Cohen’s d > 1.15; i.e. moderate size).

**Table 4:** Driving characteristics of the sample.

	Situations per person		Total sample (N = 84)	Fit to drive (n = 59)	Possibly unfit to drive (n = 25)	t value	p value	Cohen`s d**
**Distance behavior**	n = 4	mean	0.1	0.1	0.1	0.51	0.611	0.122
		(SD)	(0.2)	(0.2)	(0.3)			
	100%	percentage	1.5%	1.3%	2.0%			
**Lane keeping**	n = 13	mean	0.8	0.4	1.5	3.36	0.001	0.802
		(SD)	(1.4)	(0.9)	(2.1)			
	100%	percentage	5.9%	3.4%	11.7%			
**Communication and interaction**	n = 4	mean	0.2	0.1	0.4	3.25	0.002	0.776
		(SD)	(0.5)	(0.4)	(0.6)			
	100%	percentage	5.1%	2.5%	11.0%			
**Speed behavior**	n = 25	mean	1.5	0.9	3.0	5.12	0.000	1.222
		(SD)	(1.9)	(1.1)	(2.6)			
	100%	percentage	6.0%	3.5%	11.9%			
**Observation of blind spots**	n = 15	mean	0.8	0.3	1.9	6.93	0.000	1.654
		(SD)	(1.2)	(0.7)	(1.5)			
	100%	percentage	5.1%	1.8%	12.8%			
**Give way**	n = 17	mean	2.6	2.0	4.1	6.02	0.000	1.437
		(SD)	(1.7)	(1.2)	(1.8)			
	100%	Percentage	15.4%	11.8%	24.0%			
**Indicating/ signalling**	n = 28	mean	0.7	0.5	1.1	2.59	0.011	0.618
		(SD)	(1.0)	(0.9)	(1.1)			
	100%	Percentage	2.4%	1.8%	3.9%			
**Attentive/ perceptive behavior**	n = 12	mean	1.3	0.9	2.0	7.43	0.000	1.773
		(SD)	(0.8)	(0.5)	(0.8)			
	100%	Percentage	10.4%	7.8%	16.7%			
**Anticipatory driving**	16	mean	0.4	0.2	0.8	4.36	0.000	1.04
		(SD)	(0.7)	(0.2)	(1.0)			
	100%	Percentage	2.4%	1.2%	5.3%			
**Total errors**	134	mean	8.2	5.4	14.9	8.31	0.000	1.98
		(SD)	(6.5)	(3.4)	(7.2)			
	100%	Percentage	6.1%	4.0%	11.1%			

Mean number of errors, t-test statistics, and percentage of errors within behavioral driving-assessment categories are shown. The post-hoc grouping of the sample regarding fitness-to-drive showed signifcant differences with moderate effect sizes in nearly all observed categories, with highest scores for attention-related categories

* Independent samples t-test

** Interpretation of d according to Ferguson [36]: 0.41 = small effect; 1.15 = moderate effect; 2.70 = strong effect.

Table 5 shows the results of the fitness-to-drive tests, together with subject demographics. A post-hoc analysis showed between-group differences with small effect sizes for *age*, *gender*, *estimated kilometers driven per year*, and *number of years posessing a class B driver’s license (cars)*. No differences were found for *level of education*. Additional correlative analysis for the total sample reported elsewhere [39] revealed significant intercorrelations between a number of variables. The older the participants the lower the amount of *estimated kilometers per year* (correlation of *estimated kilometers per year* and *age r* = –0.27). *Estimated kilometers per year* is also related to *gender* (correlation of *estimated kilometers per year* and *gender r* = –0.29). Differences in the number of *estimated kilometers per year* between males and females were confirmed by the Mann-Whitney-Test (*p* = 0.007); women drove 12 224 km/year, male participants 15 716 km/year, a difference of about 3500 km/year or roughly 30%.

**Table 5:** Fitness to drive and demographics.

		Total sample (N = 84)	Fit to drive (n = 59)	Possibly unfit to drive (n = 25)	t / χ^2^	p value*	Cohen's d***
**Age (mean, SD)**		68.9 (6.6)	67.1 (5.1)	73.04 (8.05)	3.422	0.002	0.52
**Gender (n, %)**	**Male**	56 (66.7%)	44 (52.4%)	12 (14.3%)	9.313	0.002	
	**Female**	28 (3.3%)	15 (17.9%)	13 (15.5%)			
**Estimated distance driven per year in km (mean, SD)**		14409 (6805)	15932 (6849)	10500 (4963)	3.460	0.001	0.36
**Estimated distance driven per year in km (n, %)**	**0–5000**	0 (0.0%)	0 (0.0%)	0 (0.0%)	28.035**	0.000	
	**5001–10000**	28 (34.1%)	12 (14.6%)	16 (19.5%)			
	**10001–15000**	29 (35.4%)	25 (30.5%)	4 (4.9%)			
	**15001–20000**	18 (21.9%)	15 (18.3%)	3 (3.7%)			
	**> 20000**	7 (8.5%)	7 (8.5%)	0 (0.0%)			
**Number of years posessing a class B driving license (mean, SD)**		46.4 (7.6)	44.9 (6.4)	50.0 (9.1)	2.584	0.014	0.40
**Level of education (n, %)**	**ISCED Level 2**	14 (16.7%)	10 (11.9%)	4 (4.8%)	4.889	0.180	
	**ISCED Level 3**	32 (38.1%)	21 (25.0%)	11 (13.1%)			
	**ISCED Level 4**	11 (13.1%)	6 (7.1%)	5 (6.0%)			
	**ISCED Level 5**	27 (32.1%)	22 (26.2%)	5 (6.0%)			

The post-hoc grouping of the subject sample with respect to fitness-to-drive showed signifkant differences with however rather small effect sizes. T-tests were used for continuous mesasures, and χ^2^ tests of independence for categorial measures.

* Independent samples t-test used for continuous variables, and chi-square test for categorical variables (weighted by frequencies; expected values taken from the fit-to-drive group).

** n = 82 due to missing data for two subjects; cells counting zero (i.e. 0–5000 km; > 20000 km) for either expected or observed frequencies were excluded from calculation (calculation not recommended or possible for cells having fewer than five cases).

*** Interpretation of Cohen’s d following Ferguson [36]: 0.41 = small effect; 1.15 = moderate effect; 2.70 = strong effect.

### Visual assessment

Results of the visual assessment are shown in Table 6. Significant results, if of small impact on driving performance, were found for visual field size and visual acuity. The maximum horizontal visual field size (termed binocular visual field) varied, in total, between 115° and 161° but the average field size for the fit drivers was slightly larger (151°) than that for the bad drivers (144°). Visual acuity, being overall good and even at its lowest (logMAR 0.3 or 20/40, viz. 0.5) far from values that would indicate visual impairment, showed also in the mean a significant, but small, difference between the groups. For central-foveal and peripheral contrast sensitivity, no significant relationship to driving performance was found.

**Table 6:** Visual performance indicators for the participants by group.

	Total sample (N = 84)	Fit to drive (n = 59)	Possibly unfit to drive (n = 25)	t value	p value	Cohen`s d
**Visual acuity** (logMAR)	–0.01 (0.10) (0.30 to –0.10)	–0.03 (0.10) (0.30 to –0.10)	0.04 (0.11) (0.30 to –0.10)	2.71	0.008	0.647
**Binocular visual field**^1^ (deg)	149.3 (10.85) (115 to 161)	151.3 (9.3) (116 to 161)	144.4 (12.9) (115 to 157)	2.69	0.009	0.642
**Foveal R_contrast**^2^ (Michelson contrast, %)	1.96 (0.69) (0.84 to 5.24)	1.87 (0.71) (0.84 to 5.24)	2.17 (0.60) (1.22 to 3.40)	1.81	0.074	0.432
**Near-peripheral (10°) R_contrast**^2^ (Michelson contrast, %)^3^	3.53 (1.53) (0.76 to 7.53)	3.45 (1.52) (0.76 to 7.29)	3.73 (1.58) (1.51 to 7.53)	0.76	0.450	0.181

Values are presented as mean ± (SD) and range. The post-hoc grouping of the sample regarding fitness-to-drive showed signifcant differences with rather small effect sizes in nearly all observed performance categories.

* Independent samples t-test

** Interpretation of d according to Ferguson [36]: 0.41 = small effect; 1.15 = moderate effect; 2.70 = strong effect.

^1^ binocular visual field size measured in the horizontal meridian

^2^ R_contrast: recognition contrast threshold according to Strasburger [24]

^3^ Michelson contrast = 100% · (Lmax–Lmin) / (Lmax + Lmin) [40]

As a predictive analysis, multiple linear regression was used to explain the relationship between driving performance and the visual performance indicators (Table 7). To reduce the predictive model to only those variables that explain additional variance in driving performance, a stepwise regression procedure with backward elimination of variables was chosen (Wald criterion alpha: 0.1). The resulting regression model accounts for about 18% (R^2^ = 0.174, F(2, 81) = 8.51, p < 0.01) of the variance found in driving performance. Visual acuity and Binocular visual field, in summary, show significant regression weights indicating that drivers who score better on those two variables are expected to show slightly better driving performance.

**Table 7:** Multiple regression of visual performance indicators (backward elimination of variables).

	Regression coefficient B	Standard error B	Beta	t value	Sig.
**Visual acuity** (logMAR)	−2.388	0.823	−0.294	−0.901	0.005
**Binocular visual field** (degrees)	−0.21	0.008	−0.268	−2.638	0.01

The final regression model had two significant predictors.

*R* = 0.424 for step 1, *Delta R* = -0.006 for step 3, *p* < 0.10, *df* = 1, *df2* = 80

### Psychometric assessment

We repeated the procedure described above to analyze the impact of psychological (psychometric) parameters (Table 8). Seven out of ten variables revealed significant effects on driving performance, divided attention showing the most prominent effect (Cohen’s d = 1.41; i.e. moderate size). Intelligence, learning ability and reaction time showed no significant effect on driving performance.

**Table 8:** Psychological performance indicators.

	Total sample (N = 84)	Fit to drive (n = 59)	Possibly unfit to drive (n = 25)	t value	p value	Cohen`s d
**Intelligence** (AMT)	0.05 (0.95) (−2.82 to 2.06)	1.39 (1.00) (−2,82 to 2,06)	−0.17 (0.82) −2,56 to 1,32)	1.37	0.175	0.327
**Learning ability** (NVLT)	0.00 (1.00) (-2.69 to 1.91)	-0.03 (1.08) (-2.69 to 1.91)	0.08 (0.80) (-1.77 to 1.45)	0.51	0.61	0.122
**Visuo-motoric coordination** (B19)	−0.19 (1.02) (−1.44 to 3.53)	−.24 (0.79) (−1.44 to 2.29)	0.53 (1.31) (−1.25 to 3.51)	−3.34	0.001	0.797
**Perceptual performance in traffic situations** (TAVTMB)	−0.16 (0.97) (−2.53 to 2.22)	0.16 (0.84) (−2.53 to 2.22)	−0.44 (1.12) (−2.16 to 1.49)	2.71	0.008	0.647
**Reactive performance** (DT)	0.81 (0.98) (−3.04 to 2.32)	0.27 (0.88) (−1.88 to 2.32)	−0.36 (1.07) (−3.04 to 1.26)	2.77	0.007	0.661
**Reaction time** (RT)	−0.07 (0.88) (−1.72 to 2.25)	−0.16 (0.90) (−1.72 to 2.25)	0.13 (0.83) (−1.50 to 1.93)	−1.41	0.163	0.272
**Selective attention** (COG)	0.04 (0.96) (−5.00 to 1.03)	0.24 (0.76) (-2.85 to 1.03)	-0.44 (1.20) (-5.01 to 1.03)	3.13	0.002	0.747
**Selective attention** (SIGNAL)	−0.72 (0.93) (−1.44 to 4.67)	−0.25 (0.71) −1.44 to 1.72)	0.35 (1.24) (-0.81 to 4.67)	−2.77	0.007	0.661
**Divided attention** (PP DEV)	0.00 (1.03) (−1.08 to 5.99)	−0.31 (0.58) (−1.07 to 1.17)	0.73 (1.42) (−0.44 to 5.99)	−4.78	0.000	1.141
**Peripheral detection** (PP FOV)	−0.00 (1.00) (−3.24 to 1.53)	0.13 (0.93) (−3.25 to 1.53)	−0.33 (1.11) (−2.53 to 1.15)	1.97	0.052	0.470

Psychological performance indicators of the participants, by group. Variables are presented in mean ± (SD), and range. The post-hoc grouping of the sample with respect to fitness to drive showed signifcant differences with small to moderate effect sizes; highest score for divided attention. Indivudal test scores were converted into standardized z-scores, enabling readers to compare scores from different scaling systems.

* Independent samples test

** Interpretation of d according to Ferguson [36]: 0.41 = small effect; 1.15 = moderate effect; 2.70 = strong effect.

Again a multiple linear regression with backward elimination of variables was used to find the set of variables most predictive for driving performance (Table 9). The resulting regression model accounts for about 30% (R^2^ = 0.305, F(2, 81) = 17.79, p < 0.01) of variance in driving performance. Selective attention (test procedures *Cognitrone*, “COG) and divided attention (*Peripheral Perception Test*, “PP”) show significant though small regression weights indicating that drivers with better attentional skills show better driving performance.

**Table 9:** Multiple regression of psychological performance indicators.

	System output	Regression coefficient B	Standard error B	Beta	t value	Sig.
**Selective attention** (COG)	sum of correct rejections of non-congruent stimuli	−0.045	0.018	−0.236	−2.42	0.018
**Divided attention** (PP)	duration of mistakes on a central tracking task in a dual task scenario	0.075	0.017	0.432	4.43	0.000

Multiple regression of nine psychological performance indicators (backward elimination of variables). The final regression model had four significant predictors explaining the main differences in driving performance.

*R* = 0.600 for step 1, *R* = 0.552 for step 27, *Delta* = −0.020, *p* < 0.10, *df* = 4, *df2* = 80

To calculate the amount of variance of attention measures account for beyond pure vision measures, we did further regression analysis. The analysis included both visual and psychometric parameters shown to be important for driving performance in the preceding regressions (see Tables 7 and 9). The regression model shows three significant predictors of which attentional measures (selective attention, β = −0.271, p = 0.005, and divided attention, β = 0.334, p = 0.001) are more predictive than visual parameters. The mixed regression model accounts for about 40% (R^2^ = 0.388, F(4, 78) = 12.38, p < 0.01) of variance in driving performance (Table 10). Attentional measures therefore account for about 20% of variance beyond that for the vision measures (visual acuity, binocular visual field) that explained 18% of variance in driving performance (cf. visual assessment). Multicollinearity, i.e., correlations between predictors, is low (roughly 10%). Indicators for multicollinearity (tolerance and variance inflation factor (VIF)) are close to the ideal level (the obtained value of close to one, in both cases, indicates that predictors are not correlated).

**Table 10:** Multiple regression of visual and attentional performance indicators.

	System output	Regression coefficient B	Standard error B	Beta	t value	Sig.	Tolerance	VIF^1^
**Selective attention** (COG)	sum of correct rejections of non-congruent stimuli	−0.050	0.017	−0.271	−2.859	0.005*	0.870	1.15
**Divided attention** (PP)	duration of mistakes on a central tracking task in a dual task scenario	0.056	0.016	0.334	3.411	0.001*	0.818	1.22
**Visual acuity**	logMAR	−1.671	0.721	−0.212	−2.317	0.023*	0.936	1,07
**Binocular visual field**	horizontal visual field size in degrees	−0.011	0.007	−0.145	−1.561	0.123	0.915	1.09

The regression model shows three significant predictors. Attentional measures (selective attention (β = −0.271; p = 0.005) and divided attention (β = 0.334, p = .001) are more predictive than visual parameters.

*R* = 0.623, *p* < 0.05, *df1* = 4, *df2* = 78

^1^VIF = Variance Inflation Factor

*p<0.05

### Predicting driving ability by means of bivariate logistic regressions

Finally, a binary logistic regression was performed to determine the relative impact of visual and psychometric parameters as part of a combined diagnostic multilinear model predicting drivers as being fit or unfit to drive. The logistic regression model was statistically highly significant (χ^2^(4) = 28.619, *p* = 0.000 with *df* = 4). The result is in line with the result of the Hosmer-Lameshow goodness-of-fit test being not significant i.e., that the model prediction does not significantly differ from the observed values. The final model explained 42% (Nagelkerke R^2^) of the variance in fitness to drive revealing a moderate goodness of fit between the predictors and the prediction. The model correctly classified 80% of the participants providing high specificity (93%). Sensitivity was low (46%), however, identifying just 11 out of 25 bad drivers of the standardized driving test (Table 11).

**Table 11:** Predicting fitness to drive.

		Standardized driving test		
		Unfit to drive (score > 2,5)	Fit to drive (score ≤ 2,5)	Predictive values (PV)
**Test battery**	Unfit to drive (score > 2,5)	11	4	Positive PV 73.3%
	Fit to drive (score ≤ 2,5)	13	55	Negative PV 80.9%
**Diagnostic power**		Sensitivity 45.8%	Specificity 93.2%	Accuracy 79.8%

Concordance of fitness to drive predicted by a test battery of visual and psychometric tests and standardized on-road driving test. The test battery correctly identified participants rated as safe (globalized driving score ≤ 2,5) or unsafe (score > 2,5) with an accuracy of 79.8%.

*Cutoff 0*.*50*

Performance of the model rests on just two significant predictor variables. Low selective attention scores (COG) reduced the probability of being classified as fit to drive significantly (15% reduction of likelihood, Exp (B) = 0.848). Increased scores in divided attention were associated with an increased likelihood (28%, Exp (B) = 1.275) of being classified as fit to drive. None of the standard visual performance indicators (contrast sensitivity, visual acuity) added significantly to the model (Table 12).

**Table 12:** Identifying best predictors for fitness to drive.

	Regression coefficients	Wald	df	Sig.	Exp (B)	95% CI Exp (B)	
						Lower value	Upper value
**Selective attention** (COG)	−0.164	4.353	1	0.037	0.848	0.727	0.990
**Divided attention** (PP)	0.243	7.034	1	0.008	1.275	1.065	1.526
**Binocular visual field** (degrees)	−0.027	1.070	1	0.301	0.973	0.924	1.025
**Visual acuity** (logMAR)	−0.350	2.225	1	0.136	0.705	0.445	1.116

Regression coefficients, Wald statistic, Odds Ratio (Exp (B)) and 95% confidence interval (CI) for predicting fitness to drive. Attentional abilities added signifcantly to the accuracy of classification.

*Model-Chi*^*2*^ *= 28*.*619; df = 4*, *p = 0*.*000; Hosmer-Lemeshow-Chi*^*2*^ *= 5*.*978*, *df = 8*, *p = 0*.*650; Nagelkerke R*^*2*^ *= 0*.*417*

## Discussion

We investigated the relevance of visual and psychometric predictors for fitness to drive in a sample of elderly drivers. The diagnostic power of a state-of-the-art psychometric test battery on the Vienna Test System, combined with a set of standard visual parameters recommended for assessing fitness to drive by the Eyesight Working Group of the European Union (visual acuity, field of view, and letter contrast sensitivity) turned out to be surprisingly low. We thus believe the test battery, even being state-of-the art and including additional visual tests, does not satisfactorily meet its main purpose, which is screening for fitness-to-drive and thereby select bad drivers to insure traffic safety. Bad drivers in our study revealed deficits in particular in observational/attentional behavior related to traffic safety (observation of blind spots, attentive and perceptive behavior, give way) indicating below-average driving skills where the driver might fail to pass a licensing test. However, only 11 out of 25 drivers who showed impaired driving performance in the standardized on-road driving test were identified by the augmented test battery (i.e., test sensitivity was 46%). The test battery as designed would thus not even be able to appropriately screen out drivers who were most at risk for driving accidents (i.e. drivers scoring ≥3.50 or worse in the driving test). Out of seven risky drivers, three had been incorrectly labeled as “fit to drive”. In short, the diagnostic model failed to reliably predict fitness to drive.

Another main finding of the study focuses on the practical relevance of the predictors involved. Visual performance parameters required by law had no meaningful impact on driving performance. This indicates an astounding gap between mandatory regulations of state authorities and research results. Visual testing, deemed particularly important, explained only about 20% of variance in driving performance, in contrast to psychological testing which accounts for about 30%, based on mainly two single predictors: performance in selective-attention and divided-attention tests. Our results are in line with those of Staplin et al. whose aim is updating a set of screening guidelines for older drivers with the American Association for Motor Vehicle Administrators (AAMVA) [13]. The validity criterion there, unlike the criterion of driving performance in the present study, are traffic-safety outcome measures provided by the National Highway Traffic Safety Administration. The study also revealed the importance of perceptual-cognitive measures addressing selective and divided performance as potentially useful predictors. Interestingly, no exclusively visual screening test was part of the guidelines recommended by Staplin et al., in spite of driving being unarguably a highly visual task [23].

The role of vision in driver safety and driving performance was summarized by Owsley and McGwin in a comprehensive review of the literature [23]. Although driving performance should be linked to driver safety in theory, they found little empirical evidence for that link. This means that road users who demonstrate impaired driving performance in on-road driving tests are not necessarily the ones at high risk for future crash involvement, and vice versa. Notwithstanding the ubiquitous visual screening being the standard at licensing agencies for determining driving fitness, the reviewed studies mostly revealed *no significant relationship* between visual acuity and driving safety (i.e. with motor-vehicle collision involvement). This is also true for visual-acuity related driving performance decrements (e.g. sign recognition) which do not translate into reduced safety. The authors conclude that visual acuity testing does not measure the visual skills required for driving. For visual field impairment, the reviewed studies showed mixed results. Owsley and McGwin refer to a famous large-scale study (i.e., 10.000 drivers) conducted by Johnson & Keltner, which showed that drivers with severe binocular field loss had significantly higher motor vehicle collisions and violation rates compared to those without loss [41]. Similar results were found by Rubin et al. [42]. In contrast to that, several other studies found no significant relation of visual field impairment and driving safety. Owsley and McGwin interpret the ambiguous results as due to differences in the definition of visual field impairment, mixed procedures of visual field measurement and missing control of compensatory strategies (i.e., eye and head movement) [23]. Concerning contrast sensitivity there are no known licensing regulations in the U.S. or Europe that require its assessment, but numerous studies have reported significant associations between impaired contrast sensitivity and crash involvement [42, 43] or driving performance [44, 45]. The authors close their review promoting a more practical approach to improve the efficacy of vision screening in assessing fitness-to-drive by supplementing the screening of visual acuity by other types of screening approaches, focusing on contrast sensitivity, visual field, processing speed, and divided attention.

Our conclusion is similar to that stated by Owsley & McGwin [23]. As visual impairment is mostly related to eye diseases (i.e. glaucoma, age-related macular degeneration [AMD], retinitis pigmentosa, cataract) it is the assignment of ophthalmologists and optometrists to test the integrity and health of the visual system as part of a medical check-up or health-care activity. The primary objective of visual screening might be the implicit reason why some studies have found significant relationships between visual function assessment and measures of driving performance and safety, and others did not. The extent of the relationship is dependent on the nature of the sample under observation [21]. For example, population-based studies have failed to find an association between contrast sensitivity deficits and increased crash risk [42, 46], whereas studies of drivers with diagnosed cataracts (who showed a pronounced impairment of contrast sensitivity) revealed a strong relationship between contrast-sensitivity impairment and increased crash risk [43]. In sum, several studies have shown that people suffering from eye diseases might show impaired driving performance dependent on the severity of symptoms associated with eye diseases [43, 47–49]. Thus, in a health-care setting visual diagnostics are effective in revealing accompanying risks for persons concerned with eye diseases that should be taken care of. Future studies are needed to further explore the ability to drive safely with visual impairment, taking into account compensatory strategies that might help to prolong driving ability [50]. Importantly, however, visual acuity tests designed for clinical diagnosis and monitoring of eye health are not at all the choice for a screening of fitness-to-drive in a healthy population. As Wood & Owens, e.g., show, results of a visual acuity test in most cases are not at all, or only minimally, relevant for fitness to drive [51].

A screening for fitness-to-drive should reflect the complexity of the driving task; we now enter the domain of psychological testing. If scores fail the cut-off criterion, the respective drivers might be referred for further testing (e.g., to an on-road test). But how to install psychological testing in an adequate manner in licensing policies of state authorities? The European Council Directive 2006/126/EG [52] gives general recommendations for license renewal intervals that may be related to a screening for fitness to drive. As of 19 January 2013, licenses issued by Member States shall have an administrative validity of 10 or 15 years. EU Member States are free to reduce the period of administrative validity to the age of 50 years in order to allow for an increased frequency of medical checks or other specific safety measures, such as requiring attendance to refresher courses. There is widespread agreement that aging generally results in some level of decline in sensory, perceptual, cognitive, psychomotor, and physical performance, and therefore probably also in driving skills [53]. However, age is not a useful index for determining intervals for license renewal or frequency of medical checks. The arguments against age-based assessment are many and varied: it has no demonstrable road safety benefits, it prompts premature cessation of driving, and it prompts older people to use alternative modes of transportation that are riskier than the private car [53–57]. There are likely even counterproductive effects of age-based assessments, indicated by a higher rate of fatalities of older pedestrians in countries with very strict renewal requirements [58]. According to Siren et al. [59], this is also the case for an age-based cognitive screening of older drivers. After implementation of a cognitive screening program, more drivers were involved in fatal accidents attributed to a possible shift of drivers who did not pass cognitive screening to more dangerous modes of transportation (i.e. becoming pedestrians) which made them more vulnerable in traffic. There are even indications that a simple in-person license renewal may be more effective in reducing older driver deaths than tests [54]. On the other hand, independent of age there are a large number of drivers that continue driving even when they are not in condition to do so [60]. Regarding the type of experienced indisposition (physical and/or psychological), the main causes are related to physical health. Emotional or psychological shortcomings related to driving (e.g. memory problems, attentional disturbances) are either not known to the respective drivers or ignored [61]. This is especially true for drivers suffering from dementia. It is estimated that one in three dementia patients still drives [62–64]. Hence, leaving the decision to continue driving to the driver appears insufficient to ensure on-road safety. We therefore argue for a multi-tiered strategic approach for enhancing individual and public safety without, however, unjustifiedly (based on crash statistics) stigmatizing the group of older drivers as potentially risky drivers. Research has shown older drivers to be among the safest group of drivers on the road. Old people in particular need individual mobility as a significant factor for quality of life. Driving cessation is often associated with loss of independence, with isolation, and even death [56]. Hence, older drivers should drive their own car as long as possible. We therefore recommend a brief, and inexpensive, routine first-stage screening in connection with an age-independent, in-person renewal of driving licenses. Here, instruments of known validity for safe driving should be applied. Cognitive tests should cover all relevant faculties, in particular selective and divided attention in a comprehensive battery, to thereby improve test sensitivity for driving competence [65]. The routine screening should be accompanied by community policies for referring drivers there that are suspected of having a high crash risk. At the core of the referral system would be family practitioners and caregivers, trained for understanding issues of safe driving and health-related factors or illnesses that could impede fitness to drive and on-road safety [61]. Practitioners and caregivers would need to be trusted third parties, able to refer a patient to more extensive mobility checking [66].

As Baldock et al. have shown, psychological assessment is a promising way for a screening for fitness to drive [67]. The study tested 90 drivers aged from 60 to 91, and used a battery of psychological, visual, physical, and cognitive tests, combined with a standardized on-road driving test. A computerized test of visual attention, devised specifically for the study, showed itself the best predictor of on-road driving performance. Also, Wood et al. evaluated screening tests for predicting older driver performance concerning its relationship to driving safety in an on-road driving test [14, 22]. Participants included 270 [14] or 79 [22] older drivers incorporating tests from vision (e.g. static acuity, contrast sensitivity, visual fields, central motion sensitivity, central motion sensitivity), cognition (e.g. processing speed, complex reaction time for driving), and sensorimotoric domains (e.g. postural sway, total range of neck rotation). The final result of both studies is that a multi-disciplinary test battery composed of parameters derived from different domains showed a marked capacity to predict safe and unsafe driving in older adults assessed in an on-road driving test under in-traffic conditions. Similar to Baldock et al.’s study the final battery included no standard optometric visual parameters [67]. Part of the test-battery was driving exposure (kilometers driven per year), sensorimotoric assessment (Postural Sway), and cognitive testing (Dot Motion, Color Choice Reaction Time), providing a sensitivity of 80% (i.e. the proportion of unsafe drivers who were correctly identified as such by the test), and a specificity of 73% (i.e. the proportion of drivers who did not have an incident or make a critical error) [22]. Wood et al. reported similar values, 89% sensitivity and 77% specificity [14]. The levels of sensitivity and specificity reported exceed ours and those of other studies [68]. According to Wood, this might result from dissimilarities in the samples and/ or different levels of difficulty of the driving tests allowing visual or cognitive deficits to show up [22]. Methods of statistical analysis were further different. In the tradeoff between sensitivity and specificity, Wood et al. found cutoff-points by inspecting the ROC, that led to acceptable values for each slightly favoring sensitivity [14, 22, 69]. Last but not least the multi-disciplinary driving assessment proposed by Wood et al. incorporates a much wider range of domains important to driving, mirroring the underlying multi-factorial nature or driving [1, 70]. Our study focused exclusively on visual and cognitive assessment. Nonetheless, both studies come to the same conclusion, that mainly cognitive assessment provides the key predictors for safe and unsafe driving in older adults, visual assessment in comparison turns out to be less effective.

While our findings do not provide a clear guidance for predicting fitness to drive, the study results are consistent with previous research [22, 23, 67], suggesting the need for a specific test battery, with demonstrated practical relevance, for a population-based screening for safe and unsafe driving in older individuals. Towards that goal, our results clearly indicate the superior effectiveness of psychological assessment over a visual screening. Our results are based on driving assessment under real traffic conditions using a standardized route providing a wide variety of typical driving challenges. Agreement in ratings between the driving instructor and psychological supervisor were high, indicating reliable on-road driving assessment. A limitation of statistical analysis in the present study might, however, be a selection bias of participants. In Germany there is no restriction for driving licenses based on cognitive measures. Cognitive measures are required only for drivers having committed documented, repeated traffic violations (speeding, jumping red lights, etc.). Hence, the visual variability in the sample was indeed limited due to selective processes (e.g., datasets indicating ocular or visual pathway disease were excluded since persons with poor visual acuity get no driving license). In contrast, no screening for cognitive deficits took place, providing room for variability in cognitive parameters. Thus, it is fair to say that attention measures were more predictive than vision measures in a sample pre-screened for clear-cut visual deficits but these results might not generalize beyond that restriction. Furthermore drivers aware of their visual deficits or driving impairments might have not responded to our newspaper announcement inviting to a research project related to fitness-to-drive. Our sample may thus not fully represent the population of elderly drivers. Another limitation of our study is a limited number of drivers that had been rated as unfit to drive in the on-road driving test (25 out of 84). While that proportion of 30% may be representative of the elderly population, it is not the best precondition for logistic regression which works better with a balanced sample size (50%/50%) [71]. Classification accuracy, in the unbalanced situation, is not of judging model performance. The proportion of drivers rated unfit to drive in our sample was below that value (30%) meeting the recommended minimum limit (N = 25).

In summary, our study highlights the need to take notice of the disparate goals of medical diagnosis in health care and traffic safety, and distinguish screenings in the tradition of ophthalmologists and optometrists from screenings for fitness to drive addressing driving safety of road users. The former, based on visual acuity testing and assessment of the intactness of the visual field, is highly effective in clinical diagnosis and monitoring of eye diseases. The latter addresses human performance in a highly complex behavioral task: *driving*. The improved practical relevance and superior effectiveness of specific test batteries designed exclusively for that purpose has been shown, here and elsewhere. Future research should take into account ongoing innovation in vehicle technologies which will also alter requirements for the screening for fitness to drive. Advanced driver-assistance systems (ADAS), designed to minimize human error, might compensate for known driving deficits that accompany a driver’s sensory impairments underlying bad driving performance [23]. Increasing the automating of driving will shift active drivers to the role of a supervisor of the car, thus reducing the complexity of driving to that of a simple monitoring of the system [72–75]. Inherently, the pronounced shift of technology to automatic driving might shift the focus of a screening for fitness to drive even more to an assessment of attentional abilities needed to monitor the system.
